# Is There Ozone in the Atmosphere?

**Published:** 1881-11

**Authors:** 


### IS THERE OZONE IN THE ATMOSPHERE?

Two important papers on atmospheric ozone, by E. Schöne, are discussed in *Nature.* This observer, who has given much careful study to the subject of ozone, says that the smell of ozonized oxygen does not at all resemble the peculiar odor noticed after a lightning flash. The true smell of ozone is, however, frequently noticeable in ordinary air, and coming from the clothes of persons who may enter a room from the open air [in the winter. The ordinary potassium iodide papers are valueless as ozone measurers, according to Schöne. The humidity of the air and the hydroscopic character of the material from which the paper is made, largely influence the depth of color produced. Paper coated with thallous oxide is recommended as a measurer of the relative amount of "oxydizing principle" in the air. The paper is colored brown, owing to the production of thallic oxide, by ozone or hydrogen peroxide. The general conclusions are briefly these: 1. The papers are colored more deeply during the day than during the night; this difference is more apparent during the long days of the year. 2. Increased wind force causes increased coloration, because a greater amount of oxydizing substance is brought in contact with the paper during the time of exposure. 3. Cloudiness and rain especially influence the coloration; the heavier the rain the smaller the coloration of the paper. Direct determinations of hydrogen peroxide have shown that when the thallium papers are much colored this compound is present in the atmosphere in a comparatively large quantity. Herr Schöne regards the actual existence of ozone in the atmosphere as, at present, an open question.—*Lancet and Clinic*, Feb. 12, 1881.

